# Two Cases of Febrile Caloricity

**Published:** 1845-09

**Authors:** Bennet Dowler

**Affiliations:** New Orleans


### *Two Cases of Febrile Caloricity.* By Bennet Dowler, M.D., of New Orleans.

Prof. Huston,—My pursuits in a great degree prevent a continuation of my experiments, now very numerous, upon Febrile Caloricity. I beg leave to send you an outline of the *two last cases, without remarks.* July 22d, 1845. At 5 A. M., air 76°; at 5½, 76°. River, in the current, 83°; at 8, air 83°; at 10, 90°; at 11, 91°; at 5, P. M., near the sun, 98°; river, in the sun, 84⅓°.

A tanman; born in Switzerland; aged 30; a passenger in the ship Swanton, 51 days from Havre, (239 steerage;) sick 11 days with acute Typhoid; resident 6 hours; admitted July 21st, died next day, 30 minutes past noon; observations began 25 minutes after death; air of the dead house 94°; body finely proportioned, free from emaciation, and continued every where flexible, even in the neck, during the experiments, which lasted two and a half hours, and were witnessed by Mr. Compton, student in the Hospital, being varied in the order following every 5 to 10 or 15 minutes: Axilla, 109°, 110°, 110½°, 110¾°; rectum, 111½°, 111½°, 111½°; axilla, 110½°, 110½°; rectum, 111½°, 111½°; axilla, (in 15 m.,) 110¾°; rectum, 111°. Epigast., 109°; right epigast., 110½°; left, 110°; umbilical, 110°. The thigh was now punctured; in 2 m., 105°; when the burial interrupted the observations.

